# Orthogonally Dispersed Spectroscopic Single‐Molecule Localization Microscopy

**DOI:** 10.1002/nap2.70027

**Published:** 2026-01-29

**Authors:** Jun Lu, Lei Xu, Zhenyao Zhao, Biqin Dong

**Affiliations:** ^1^ College of Biomedical Engineering Yiwu Research Institute Fudan University Shanghai China

**Keywords:** orthogonal dispersion, single‐molecule localization microscopy, spectroscopy, super‐resolution imaging

## Abstract

Spectroscopic single‐molecule localization microscopy (sSMLM) simultaneously acquires both spatial and spectral information from fluorescent molecules, facilitating molecular characterization analysis and multicolor imaging. However, this technique poses a fundamental dilemma: a finite photon budget must be split between localization and spectroscopy, limiting the performance of both. To alleviate this trade‐off, we propose orthogonally dispersed sSMLM (ODsSMLM). By modulating single‐molecule emission spectra through an orthogonal structure, ODsSMLM allows all photons to be used for both localization and spectral characterization. Crucially, this approach provides isotropic lateral localization precision, effectively removing the inherent dispersion‐dependent localization artifacts of other methods. Using simulated data, we demonstrate that under a 3000‐photon budget, ODsSMLM attains a localization precision of 10 nm and a spectral precision of 1.1 nm. Moreover, in dual‐color imaging experiments of microtubules and clathrin, ODsSMLM achieved isotropic lateral resolution of 27 nm.

## Graphical abstract

Orthogonally dispersed spectroscopic single‐molecule localization microscopy (ODsSMLM) encodes molecular positions via two orthogonal spectral channels, enabling full photon utilization for simultaneous spatial localization and spectral analysis. By synthesizing location information from the non‐dispersive directions, it effectively eliminates the impact of spectral heterogeneity on localization.

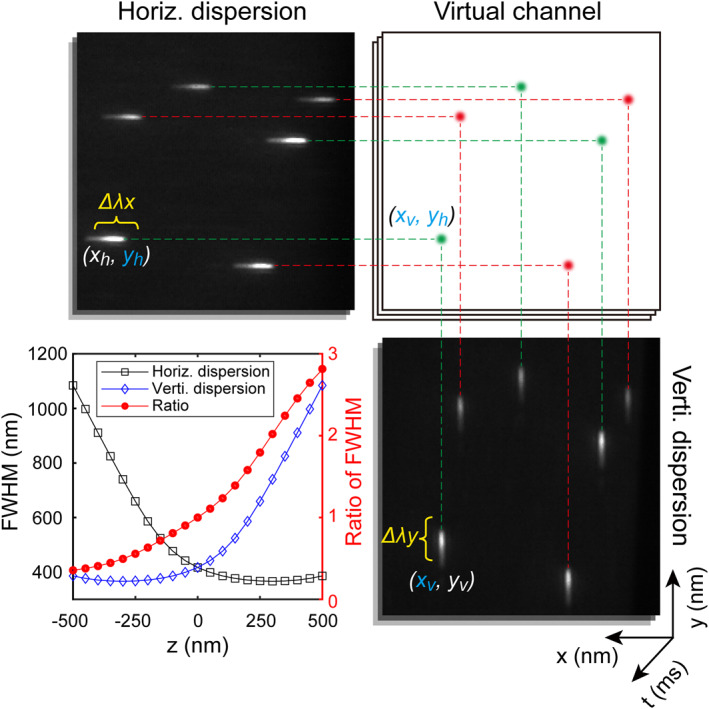

## 1 Introduction

Spectroscopic single‐molecule localization microscopy (sSMLM) exhibits outstanding spatial resolution and molecular identification capability, making it widely applicable in biological research and molecular analysis [1, 2, 3, 4, 5, 6, 7, 8]. Conventional sSMLM implementations, whether prism‐based [1, 2, 3, 4] or grating‐based [5, 6, 7, 8], inherently split the limited photon budget between independent spatial localization and spectral analysis channels. This creates a fundamental trade‐off: improving spatial precision by allocating more photons to the localization channel comes at the expense of spectral precision, and vice versa. Methods such as dual‐wedge prism‐based sSMLM aim to maximize photon throughput by minimizing optical losses [9, 10], and deep learning approaches show promise for spectral denoising [11, 12], information extraction [13], and molecular classification [8, 14]. However, these strategies still operate within the fundamental photon‐allocation trade‐off that intrinsically limits simultaneous spatial and spectral precision. Although dual‐objective sSMLM [1, 2, 3, 15, 16] maximizes photon collection through 4π imaging, it entails substantial optical complexity and constraints on sample compatibility. In contrast, low‐dispersion sSMLM [17] and color‐glass‐filter‐based sSMLM [18] achieve high photon efficiency and support high‐density imaging, but at the expense of spectral resolution, thereby limiting their applicability to functional imaging based on spectral fingerprints.

In conventional sSMLM, molecular localization using individual spectral channel is challenging. This is because the spectral heterogeneity [3, 6, 19] introduces random fluctuations along the dispersion direction, which preclude its use for precise lateral localization. To improve photon efficiency, 3D biplane sSMLM [20] creates an optical path difference between the spatial and spectral channels. This allows the spectral channel to be used for axial localization, a method that has proven superior to astigmatism‐based sSMLM [1, 21] which uses a cylindrical lens. However, this method’s lateral localization relies solely on the spatial channel, with spectral analysis performed independently. Other methods based on grating [22] or prisms [23] employ symmetric spectral dispersion to mitigate random fluctuations caused by spectral heterogeneity, thereby demonstrating superior localization precision and spectral precision. Despite this, it suffers from a reduced signal‐to‐noise ratio (SNR) in the unfolded spectral signals and struggles to eliminate localization errors along the dispersion direction, resulting in an anisotropic lateral resolution.

To fully utilize all photons for simultaneous spatial localization and spectral analysis, we propose orthogonally dispersed sSMLM (ODsSMLM), which encodes the spatial positions of fluorescent molecules by two orthogonally dispersed spectral channels. By synthesizing location information from the non‐dispersive directions, ODsSMLM effectively eliminates the impact of spectral heterogeneity on localization. Using simulated data, we demonstrate that ODsSMLM achieves isotropic spatial localization precision of 10 nm and a spectral precision of 1.1 nm under a 3000‐photon budget at dispersion value of 6 nm/pixel. In simulated dual‐color structures, ODsSMLM reduces crosstalk by 85% compared to sSMLM, while improving Fourier ring correlation (FRC) resolution by 46% compared to SDsSMLM. Moreover, we experimentally validate the method, achieving an isotropic lateral resolution of 27 nm in dual‐color imaging.

## 2 Experimental Setup for ODsSMLM

**FIGURE 1:**
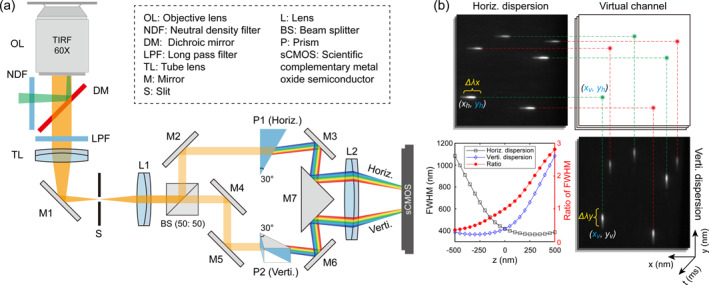
Principle and setup of ODsSMLM. (a) Optical configuration of the home‐built microscope, featuring separate paths for horizontal and vertical dispersion. (b) Illustration of the localization principle: raw images consist of horizontally and vertically dispersed spectra; a virtual channel is synthesized from the orthogonal channels to correct spectral heterogeneity and achieve robust lateral localization, whereas axial localization is determined via a biplane‐based calibration curve.

The schematic diagram of the home‐built ODsSMLM system is shown in Figure 1a. An excitation laser (FC‐405/488/556/642‐CC32600, CNI) is reflected by a dichroic mirror (Di01‐T405/488/568/647, Semrock) into a silicone immersion objective (UPLSAPO60XS2, Olympus). The emitted fluorescence is collected by the same objective, passes through the dichroic mirror and a quad‐band emission filter (BLP01‐405R/488R/561R/647R, Semrock), and is focused onto an aperture (SP40, max. aperture 7 mm × 7 mm, OWIS) by a tube lens (SWTLU‐C, Olympus) with focal length of 180 mm. The light then enters an infinity‐corrected relay system (lenses L1, L2), where a 50:50 beam splitter separates it into two optical paths. One path passes through a horizontally oriented dispersing prism (P1, 30°–60°–90° Littrow, Edmund) with dispersion angle of 30° to generate horizontally dispersed spectrum, whereas the other passes through an identical, vertically oriented prism (P2) to generate vertically dispersed spectrum. Finally, relay lens L2 focuses both orthogonally dispersed spectra onto a scientific complementary metal oxide semiconductor (sCMOS) camera (Dhyana 400BSI V3, Tucsen) for detection.

Fluorescent beads (T7279‐440/515/560/605‐100 nm, ThermoFisher) are employed to obtain orthogonal spectra for localization calibration. Figure 1b illustrates the localization principle of ODsSMLM. For the horizontally dispersed spectral channel, we integrate the spectral signals along dispersion direction to obtain the spectral profile, and calculate its centroid as coordinate $x_{h}$. Then, we integrate the spectral signals along non‐dispersion direction and perform one‐dimensional (1D) Gaussian fitting on the resulting profile, and the centroid position of the Gaussian function is assigned as coordinate $y_{h}$. Similarly, coordinates $\left(x_{v},y_{v}\right)$ of the vertically dispersed channel can be obtained. Because of spectral heterogeneity, $x_{h}$ and $y_{v}$ present random fluctuations along the dispersion axes. Thus, we can adopt the non‐dispersion coordinates $\left(x_{v},y_{h}\right)$ as the molecular position. Critically, the positional offsets induced by spectral fluctuations are theoretically identical in both channels, which allows refinement of the molecular position, as defined by Equations (1) and (2):

(1) $$\begin{array}{c} \Delta \lambda_{x}=\left|x_{h}-x_{v}\right|, \end{array}$$

(2) $$\begin{array}{c} \Delta \lambda_{y}=\left|y_{v}-y_{h}\right|, \end{array}$$

where $\Delta \lambda_{x}$ and $\Delta \lambda_{y}$ are positional offsets in horizontal and vertical channels. In experimental imaging system, the average value Δλ can be taken as the actual offset.

When $x_{h}<x_{v}$ and $y_{v}<y_{h}$, the average offset Δλ is defined by Equation (3):

(3) $$\begin{array}{c} \Delta \lambda =\frac{\Delta \lambda_{x}\;+\;\Delta \lambda_{y}}{2}=\frac{x_{v}\;-\;x_{h}\;+\;y_{h}\;-\;y_{v}}{2}, \end{array}$$

and, the refined coordinates (*x*, *y*) can be defined by Equations (4) and (5):

(4) $$\begin{array}{c} x=\frac{x_{h}\;+\;\Delta \lambda \;+\;x_{v}}{2}=\frac{{3x}_{v}\;+\;x_{h}\;+\;y_{h}\;-\;y_{v}}{4}, \end{array}$$

(5) $$\begin{array}{c} y=\frac{y_{v}\;+\;\Delta \lambda \;+\;y_{h}}{2}=\frac{{3y}_{h}\;+\;y_{v}\;+\;x_{v}\;-\;x_{h}}{4}, \end{array}$$

which integrate all coordinates for position calculation.

When $x_{h}>x_{v}$ and $y_{v}>y_{h}$, the average offset Δλ is defined by Equation (6):

(6) $$\begin{array}{c} \Delta \lambda =\frac{\Delta \lambda_{x}\;+\;\Delta \lambda_{y}}{2}=\frac{x_{h}\;-\;x_{v}\;+\;y_{v}\;-\;y_{h}}{2}, \end{array}$$

then, the refined coordinates (*x*, *y*) can be defined by Equations (7) and (8):

(7) $$\begin{array}{c} x=\frac{x_{h}\;-\;\Delta \lambda \;+\;x_{v}}{2}=\frac{{3x}_{v}\;+\;x_{h}\;+\;y_{h}\;-\;y_{v}}{4}, \end{array}$$

(8) $$\begin{array}{c} y=\frac{y_{v}\;-\;\Delta \lambda \;+\;y_{h}}{2}=\frac{{3y}_{h}\;+\;y_{v}\;+\;x_{v}\;-\;x_{h}}{4}. \end{array}$$

The optical components in two channels are placed asymmetrically, thus naturally forming a biplane configuration in the system, which facilitates axial localization. By integrating spectral signals along the non‐dispersion direction and performing 1D Gaussian fitting on the resulting profile, we obtained the full‐width at half‐maximum (FWHM) of the Gaussian function. Calibration curves were generated by calculating the FWHM for both horizontally and vertically dispersed spectra at various depths. We utilize the calibration curve within −500 to 500 nm for effective axial localization calibration, as shown in Figure 1b. The workflow for localization calibration under misalignment conditions is illustrated in Supporting Information S1: Note 1.

## 3 Results and Discussion

### 3.1 Localization Precision Analysis

**FIGURE 2:**
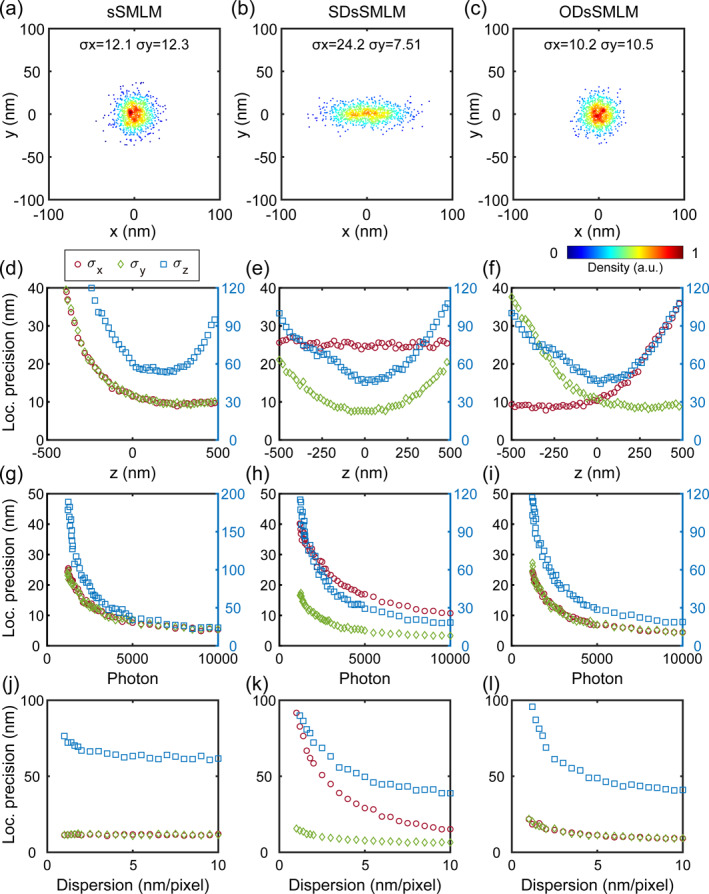
Comparison of localization precision for three methods. (a–c) Scatter plots of 1000 localizations for sSMLM, SDsSMLM and ODsSMLM at *z* = 0 nm, respectively. (d–f) Localization precision in the depth range of −500 to 500 nm. (g–i) Localization precision at *z* = 0 nm with photon number from 500 to 10,000. (j–l) Localization precision in the spectral dispersion range of 1–10 nm/pixel at *z* = 0 nm depth.

To compare the spatial localization capability of sSMLM, SDsSMLM, and ODsSMLM, we introduced numerical simulations and calculated the localization precision. For spectral generation, we first generated theoretical 3D PSFs [24], which were then convolved with the spectral profile of Alexa Fluor 647 (AF647) to yield ideal spectrum. To realistically simulate experimental conditions on camera, we sequentially superimposed shot noise and readout noise [25] onto the spectrum to generate the final simulated spectral images. The simulated spectra were subjected to different conditions such as depth, photon number, and dispersion value. The 1:3 photon allocation ratio is adopted in most published literature [5, 7, 20, 22, 26]. Because, dispersion degrades the SNR of the spectral image, necessitating a greater photon allocation for spectral channel to ensure reliable spectral information extraction. We adopted photon allocation ratio of 1:3 for conventional sSMLM, whereas both SDsSMLM and ODsSMLM employed ratio of 1:1. We compared the localization performance of the three methods with 1000 localizations at *z* = 0 nm and spectral dispersion of 6 nm/pixel, as shown in Figure 2. Figure 2a–c sequentially displays the localization scatter plots obtained by sSMLM, SDsSMLM, and ODsSMLM, respectively. We quantified the localization precision across a depth range of −500 to 500 nm, as presented in Figure 2d–f. In the sSMLM method, unequal photon assignment causes asymmetry in axial localization precision and the focus shifts in the spatial channel lead to asymmetry in lateral localization precision, as shown in Figure 2d. In simulated spectral data, the spectral centroids exhibit minimal variation across different depths. Consequently, the localization precision of SDsSMLM in the *x*‐direction demonstrates negligible depth‐dependent fluctuations (Figure 2e). At *z* = 0 nm with a spectral dispersion of 6 nm/pixel, we quantified the 3D localization precision across photon counts ranging from 500 to 10,000, as shown in Figure 2g–i. SDsSMLM exhibited inferior localization precision along the dispersion axis compared to the non‐dispersion axis, whereas both sSMLM and ODsSMLM demonstrated isotropic precision in lateral dimensions.

Furthermore, at depth of *z* = 0 nm, we systematically evaluated the localization precision with varying spectral dispersion levels (1–10 nm/pixel), as shown in Figure 2j–l. The anisotropic localization precision of SDsSMLM became particularly pronounced at lower dispersion values. All three methods achieved comparable axial precision since they shared identical biplane calibration procedures along the non‐dispersion axis.

### 3.2 Dual‐Color Imaging of Simulated Structures

To further conduct comparative analysis on reconstructed images, we simulated microtubule and mitochondrial structures that labeled with AF647 and CF660C dyes, respectively, for dual‐color super‐resolution imaging. The simulated structures were generated by the SuReSim [27] software tool. We employed total signal photon of 5000 and total background photon of 10,000 for per single blinking event. In sSMLM, the photon ratio between the spatial and spectral channels is 1:3, whereas both SDsSMLM and ODsSMLM with photon ratio of 1:1. We sequentially simulated spectra with dispersion values of 4, 6, and 10 nm/pixel, respectively.

**FIGURE 3:**
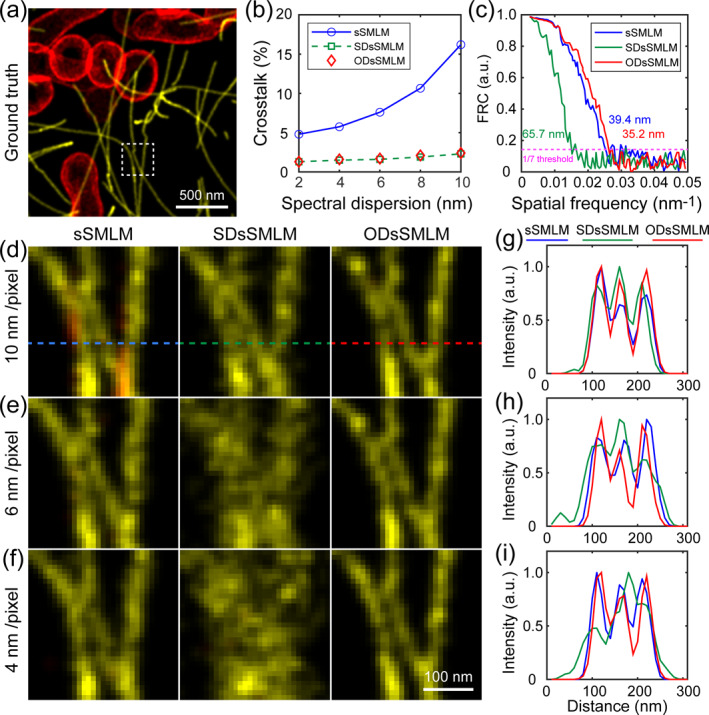
Dual‐color imaging of simulated microtubules and mitochondria structures. (a) The reconstructed dual‐color image of the ground truth structures. (b) Crosstalk of spectral centroids at spectral dispersion of 2–10 nm/pixel. (c) The FRC curve of reconstructed image. (d) Magnified images for three methods at dispersion value of 10 nm/pixel, (e) at 6 nm/pixel, and (f) at 4 nm/pixel. (g–i) Intensity profiles at position indicated by the dashed line.

Figure 3a shows the ground truth structure, which we reconstructed using three different methods for comparative analysis. The proportion of fluorescent molecules that misclassified into incorrect color channels is defined as crosstalk [1]. The crosstalk calculated at dispersion value of 2–10 nm/pixel is shown in Figure 3b. For sSMLM method, spectral extraction depends on the precision and accuracy of spatial localization. When 25% photons are used for spatial localization, the spectral centroids of the two dyes exhibit crosstalk from 4.8% to 16.2%. SDsSMLM and ODsSMLM methods combined all photons for spectral centroid calculation, so the spectra exhibit minimal crosstalk below 3%.

The FRC analysis [28, 29] was introduced to quantify imaging resolution, and the results show that ODsSMLM achieves FRC resolution of 35.2 nm, outperforming sSMLM of 39.4 nm and SDsSMLM of 65.7 nm, as shown in Figure 3c. We enlarged the reconstructed image selected by the white box, as shown in Figure 3d–f. For sSMLM, spatial localization and spectral analysis were conducted independently. Therefore, the spatial resolution was unaffected by the dispersion values. Molecular misclassification from spectral centroid crosstalk is pronounced at a dispersion of 10 nm/pixel, whereas it weakens significantly at lower dispersion values. For SDsSMLM, lateral resolution degraded at a lower dispersion value of 4 nm/pixel, due to the increased localization precision errors along the dispersion direction caused by spectral unfolding. Although SDsSMLM achieves superior molecular classification, its imaging resolution gradually deteriorates with reduced dispersion values. In contrast, ODsSMLM demonstrates excellent molecular classification and maintains isotropic resolution regardless of dispersion conditions. We further plotted intensity profiles along dashed lines, as shown in Figure 3g–i. At dispersion value of 4 nm/pixel, ODsSMLM achieves a resolution of 21 nm, outperforming sSMLM of 27 nm and SDsSMLM of 53 nm. The reconstructed 3D super‐resolution images are presented in Supporting Information S1: Figure S6.

### 3.3 Experimental Dual‐Color Imaging of ODsSMLM

**FIGURE 4:**
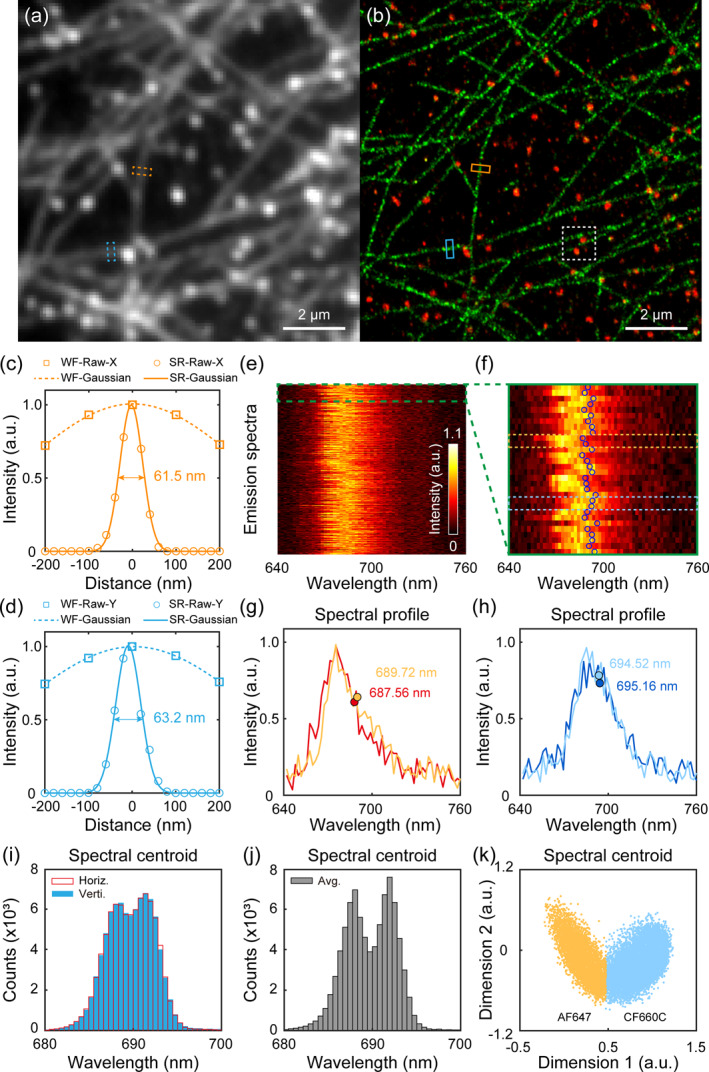
Experimental dual‐color imaging of microtubules and clathrin by the ODsSMLM method. (a) The diffraction‐limited image. (b) The reconstructed dual‐color super‐resolution image. (c, d) Intensity profiles of selected microtubule sections. (e) Extracted single‐molecule spectra in the write box. (f) Magnified image of the averaged spectra. (g, h) Intensity profiles of AF647 and CF660C spectra. (i, j) The distribution of spectral centroid for horizontal dispersion (Horiz.) spectra, vertical dispersion (Verti.) spectra, and averaged (Avg.) spectra. (k) PCA and k‐means classification results for all collected spectra.

To demonstrate the imaging capability of the ODsSMLM method for cellular structures, we performed dual‐color imaging using AF647‐labeled microtubules and CF660C‐labeled clathrin. Figure 4a shows the diffraction‐limited wide‐field image with a field‐of‐view (FOV) of 11 μm × 11 μm. The reconstructed dual‐color image (green‐microtubules and red‐clathrin) by the ODsSMLM method is shown in Figure 4b, and a larger FOV of 40 μm × 40 μm is provided in Supporting Information S1: Figure S2. The super‐resolution images with separated color channels are presented in Supporting Information S1: Figure S3. We draw the intensity profiles of selected microtubule sections for resolution analysis, and ODsSMLM exhibits isotropic resolution of 26.5 nm in *x*‐direction and 28.2 nm in *y*‐direction, as shown in Figure 4c,d. We extracted the 300 averaged spectra (Figure 4e) for display and magnified the top 30 spectra (Figure 4f), then extracted the marked spectrum and plotted their profiles and centroids, as shown in Figure 4g,h. The averaged spectra of the two dyes reveal a slight shift in spectral centroid, which is attributable to spectral heterogeneity. The separated spectra from two structures are presented in Supporting Information S1: Figure S4.

Compared with horizontal dispersion spectra and vertical dispersion spectra, the averaged spectra show a distinguishable centroid distribution, as shown in Figure 4i,j. This is because the averaged spectrum exhibits an enhanced signal‐to‐noise ratio, thereby revealing a distinct difference in spectral centroid. We employed PCA and k‐means for dimensionality reduction and clustering analysis of spectral data, yielding classification results superior to spectral centroid approach, as shown in Figure 4k. These results demonstrate that ODsSMLM possesses isotropic spatial localization capability and spectral analysis capability, experimentally enabling dual‐color cellular structure imaging.

## 4 Conclusions

Although the high spectral resolution conditions used in ODsSMLM enhance the functional imaging capabilities of sSMLM, they also reduce the signal‐to‐noise ratio of the spectral images. This makes the effective extraction of spectral information crucial. Although ODsSMLM requires sparse emission for accurate localization, it probes spectral information in two orthogonal dimensions, which may facilitate the positional analysis of overlapping spectra by exploiting their complementary features, as illustrated in Supporting Information S1: Note 2. Our current spectral analysis relies on simple signal superposition and PCA classification. Future work incorporating more advanced deep learning methods is expected to yield superior extraction and classification performance. In addition, we will explore more integrated and compact system configurations to minimize photon transmission losses and optical aberrations, thereby enabling large FOV sSMLM imaging. Furthermore, a theoretical extension of ODsSMLM to 4π imaging is conceivable, which could potentially double photon collection and enhance information‐retrieval efficiency.

In conclusion, we present ODsSMLM, a novel method that simultaneously enhances both localization precision and spectral precision. Under simulated conditions of 3000 photons and 6 nm/pixel dispersion, ODsSMLM achieved isotropic lateral localization precision of ∼10 nm. Using simulated dual‐color biological structures, we confirm that ODsSMLM reduces crosstalk by 85% compared to sSMLM, while improving FRC resolution by 46% compared to SDsSMLM. Furthermore, ODsSMLM maintains isotropic spatial resolution, and we experimentally demonstrated the spectral analysis and dual‐color imaging capabilities of ODsSMLM. We believe the proposed method is promising for high‐resolution multi‐color and functional super‐resolution imaging, with broad potential applications in live‐cell microscopy and nanoscale biological studies.

## Author Contributions

**Jun Lu:** conceptualization, methodology, validation, writing – original draft, software. **Lei Xu:** resources, data curation. **Zhenyao Zhao:** resources, data curation. **Biqin Dong:** conceptualization, writing – review and editing, project administration, supervision, resources, funding acquisition.

## Funding

This study was supported by the National Key Research and Development Program of China (Grant No. 2022YFF0708700); Shanghai Basic Research Special Zone Program (Grant No. 22TQ020); and Medical Engineering Fund of Fudan University (Grant No. yg2025‐key‐4).

## Conflicts of Interest

B.D. is a founder and equity holder of MicroLux (Shanghai) Intelligent Science & Technology Co. Ltd. and Lishi Intelligent Science & Technology (Shanghai) Co. Ltd. All other authors declare no competing interests.

## Supporting information


Supporting Information S1

## Data Availability

The source code and dataset for ODsSMLM will be publicly available at https://github.com/FDU‐donglab/ODsSMLM.
