# Supplementary material for: Orthogonally Dispersed Spectroscopic Single‐Molecule Localization Microscopy
Source: Nanophotonics. 2026 Jan 29;15(3):e70027. doi: 10.1002/nap2.70027 (PMC12965006; doi:10.1002/nap2.70027)
Supplement: Supplementary file 1 — Supporting Information S1 [file NAP2-15-e70027-s001.pdf]

Supplementary Information for

Orthogonally dispersed spectroscopic single-molecule localization microscopy

Jun Lu, Lei Xu, Zhenyao Zhao and Biqin Dong*

College of Biomedical Engineering, Yiwu Research Institute, Fudan University, Shanghai 200433, China

*Corresponding author: *dongbq@fudan.edu.cn*

Supplementary Note 1. Calibration and localization procedures.

Supplementary Note 2. Localization under three conditions using ODsSMLM.

Supplementary Note 3. 3D reconstruction and rendering.

Figure S1. Orthogonal spectral images for localization calibration.

Figure S2. Experimental dual-color imaging using ODsSMLM.

Figure S3. Super-resolution images with separated color channels.

Figure S4. Separated spectra from two structures.

Figure S5. Localization under three conditions using ODsSMLM.

Figure S6. Reconstructed 3D super-resolution images.

Supplementary Note 1

Calibration and localization procedures

Our orthogonal spectral module is modular and pluggable, thereby enabling flexible switching of the optical path between spatial and spectral channels. To achieve robust imaging under misalignment conditions, we implemented the following calibration and localization procedures.

The PSF image of fluorescent beads was first acquired in the spatial channel. After inserting the spectral module, the corresponding orthogonal spectral images were obtained. We then applied an affine transformation to establish the mapping matrices from the orthogonal spectral channels to the spatial channel: T_h for the horizontal spectral channel and T_v for the vertical spectral channel, as shown in Figure S1.

For each single-molecule emission event, the coordinate along the non-dispersive axis was determined via Gaussian fitting, while the coordinate along the dispersive axis was obtained by calculating the spectral centroid. The coordinates from both channels were then mapped to a common virtual spatial channel using the affine transformation matrices T_h and T_v established during the calibration procedure to enable pairing. The resulting paired coordinates were subsequently processed using the localization algorithm described in Section 2 to obtain the final precise coordinates.

Supplementary Note 2

Localization under three conditions using ODsSMLM

The ODsSMLM method requires sparse emission of fluorophores to ensure accurate molecular localization. However, its key advantage lies in orthogonal spectral detection: emitters overlapping in the horizontal dispersion channel may remain resolvable in the vertical channel, and vice versa. The coordinate of overlapping spectra along the non-dispersive axis serves as a common reference shared by their complementary spectral channels. Taking the overlapping case in Figure S5b as an example, we first integrate spectrum along non-dispersive axis. The resulting intensity profile [Figure S5b(IV)] is fitted with a Gaussian function to determine peak coordinates. Since the spectra overlap in the horizontal dispersion channel, this common y-coordinate is shared with their distinct x1 and x2 positions obtained from the vertical dispersion channel, yielding the final coordinates (x1, y) and (x2, y). Following the same principle, Figure S5c yields the coordinates (x, y1) and (x, y2). The characteristics of molecules can be analyzed using non-overlapping spectra.

Supplementary Note 3

3D reconstruction and rendering

We performed 3D reconstruction of the simulated structure displayed in Figure 3a. The simulation was configured with a total photon budget of 5,000. The photon allocation ratio was set to 1:3 for sSMLM, and 1:1 for both SDsSMLM and ODsSMLM. The spectral channels were configured with a total background of 10,000 photons and a dispersion value of 6 nm/pixel. We performed 3D reconstruction of the simulated dual-color structure using three methods, with a color-coded depth range from -500 nm to 500 nm, as shown in Figure S6.

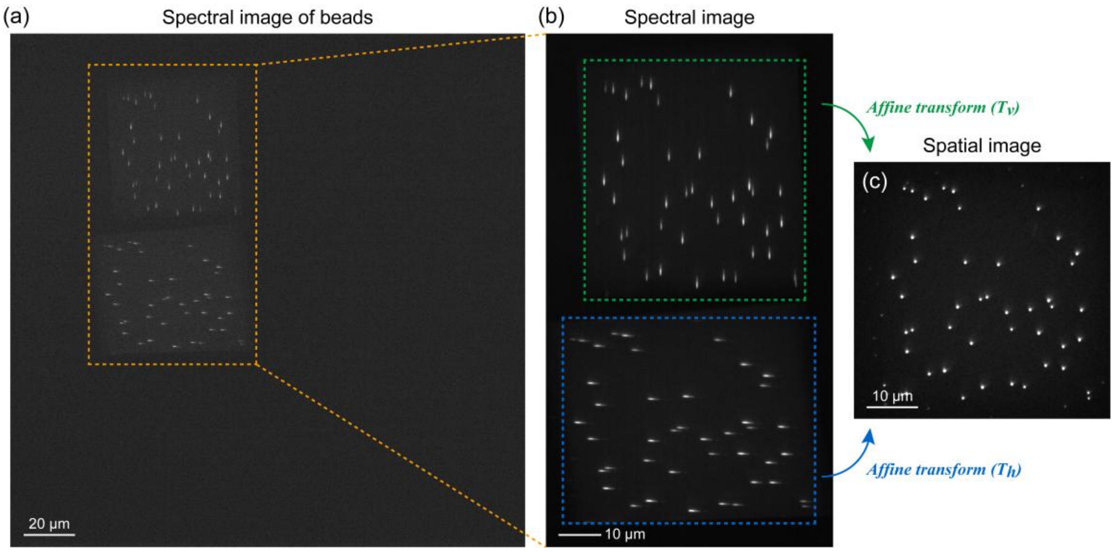

Figure S1: Using fluorescent beads for calibration between two orthogonal spectral images. (a) A raw frame of orthogonal spectral image captured by camera, with a resolution of 2048 × 2048 pixels and a pixel size of 100 nm. (b) The corresponding orthogonal spectral image and (c) the spatial image used to achieve the affine transformation.

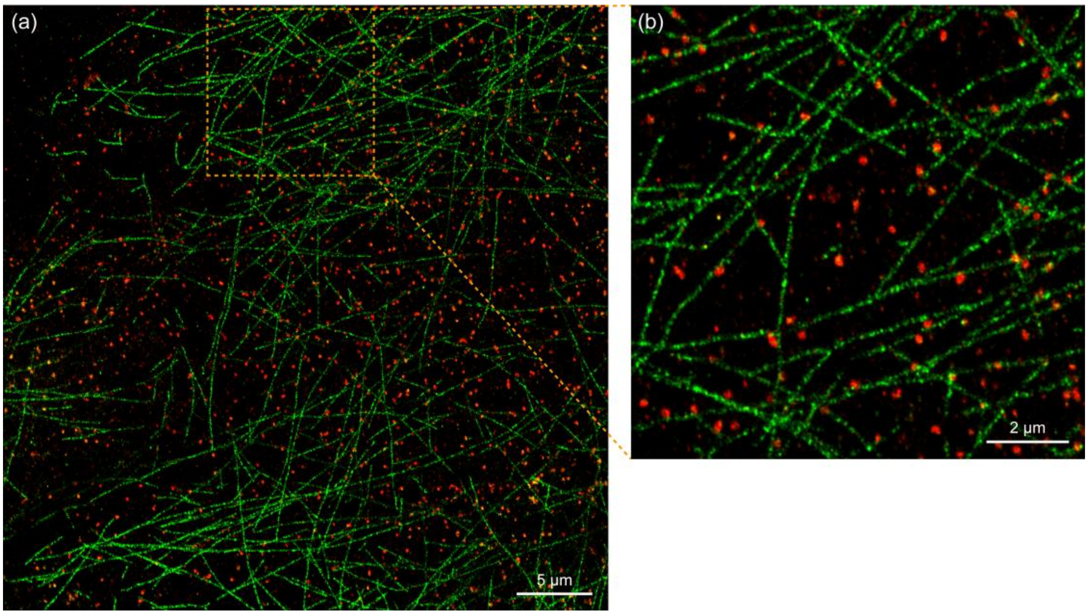

Figure S2: Experimental dual-color imaging of microtubules and clathrin using the ODsSMLM method. (a) Reconstructed dual-color super-resolution image over a 40 μm × 40 μm FOV. (b) Cropped region indicated by the dashed box in (a), showing an 11 μm × 11 μm FOV.

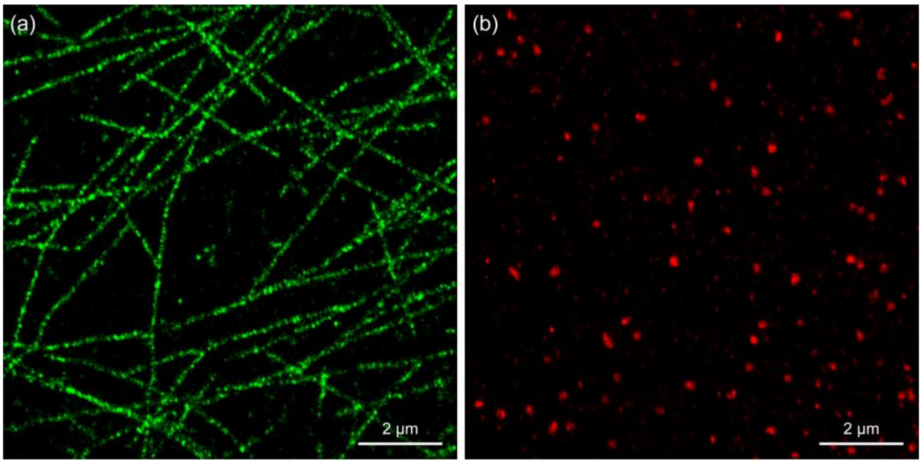

Figure S3: The super-resolution images with separated color channels. (a) microtubules, and (b) clathrin.

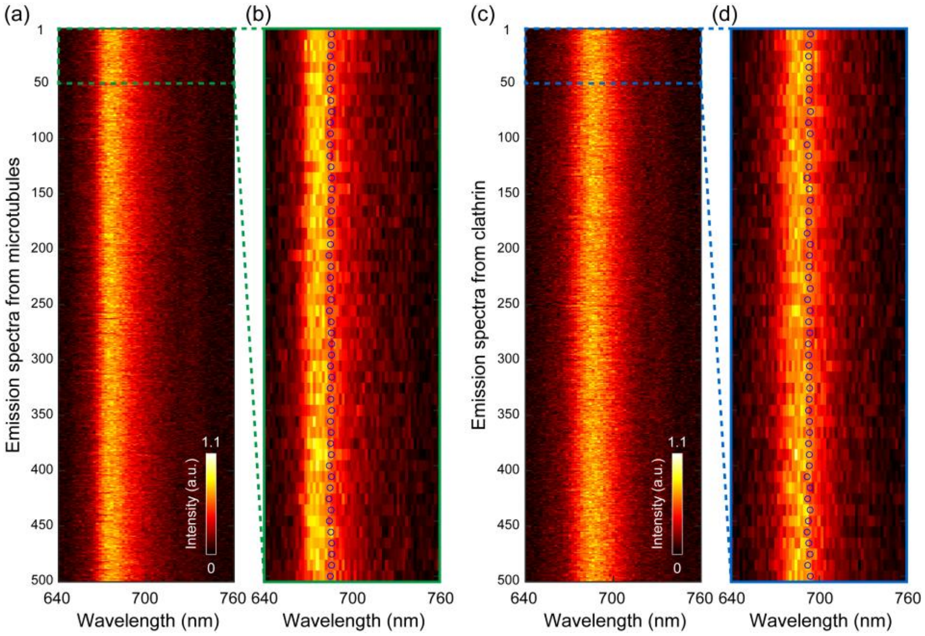

Figure S4: Separated spectra from two structures. (a) 500 emission spectra from microtubules, with the first 50 spectra enlarged in (b). (c, d) Corresponding emission spectra from clathrin.

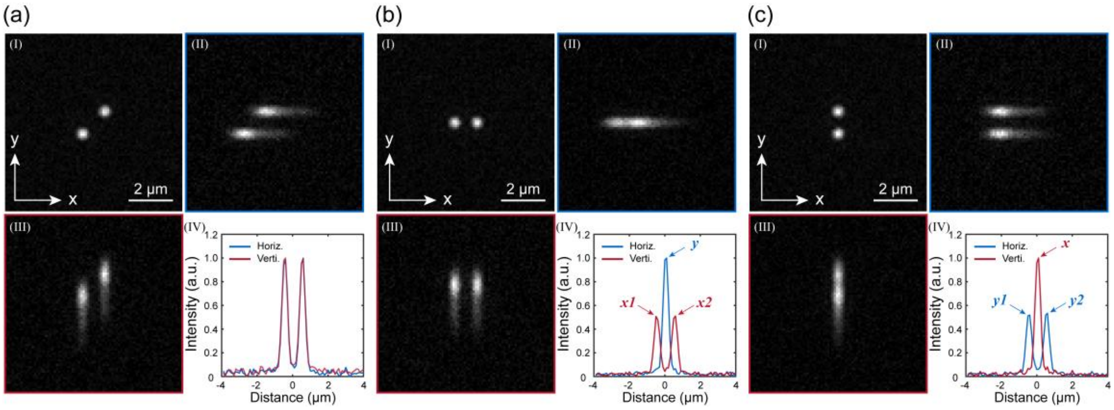

Figure S5: Localization of molecules under three conditions using ODsSMLM. (a) without overlap, (b) dispersion spectra overlapping in the horizontal direction, and (c) dispersion spectra overlapping in the vertical direction.

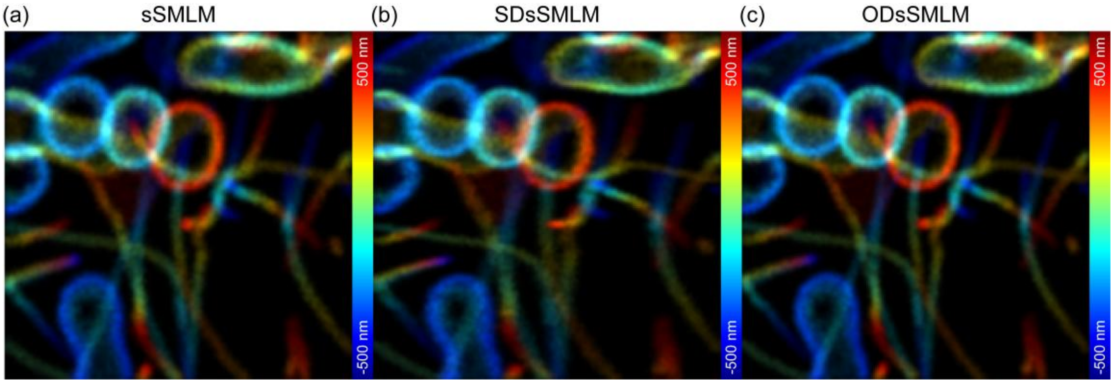

Figure S6: Reconstructed 3D super-resolution images of the simulated dual-color structure, with color-coded depth ranging from -500 nm to 500 nm. Images were reconstructed using (a) sSMLM, (b) SDsSMLM, and (c) ODsSMLM, respectively.
